# Antecedents and Outcomes of Parental Homework Involvement: How Do Family-School Partnerships Affect Parental Homework Involvement and Student Outcomes?

**DOI:** 10.3389/fpsyg.2019.01048

**Published:** 2019-05-09

**Authors:** Swantje Dettmers, Sittipan Yotyodying, Kathrin Jonkmann

**Affiliations:** Department of Educational Psychology, Faculty of Psychology, FernUniversität in Hagen, Hagen, Germany

**Keywords:** homework, parental involvement, family-school communication, achievement, well-being

## Abstract

Recent studies have demonstrated that parental homework involvement may not always foster students’ desired school outcomes. Such studies have also concluded that the quality of parental homework involvement matters, rather than the quantity. Most importantly, previous studies have shown that strong family-school partnerships (FSPs) may help to improve parental involvement. However, there is little research on how FSP is related to homework involvement. The aim of the present study is to examine the link between an effective family-school communication (EFSC) – as one aspect of FSP – and the quality of parental homework involvement in the German context. For this purpose, we developed a new measure of EFSC. Taking a self-determination theory perspective on parental need support, the quality of parental homework involvement was differentiated into two dimensions of parental *supportive* behavior: autonomy support and competence support. We analyzed the data of 309 parents (82% mothers) of school students (52% girls) who participated in an online survey. The structural equation model revealed a positive relation between EFSC and the quality of parental homework involvement, which in turn was positively associated with school performance and well-being. Moreover, we found that the quality of parental homework involvement mediated the relations of EFSC with achievement and well-being. The results of our study highlight the role of EFSC as a key performance factor that helps to improve the quality of parental homework involvement, thereby promoting student achievement and well-being.

## Introduction

Across the globe, students are set homework assignments on a regular basis since homework is generally believed to improve achievement (Paschal et al., 1984; Cooper, 1989). In their meta-analysis of school effectiveness studies, Scheerens and Bosker (1997) found a mean effect size across 13 studies of Zr = 0.06 (Fisher’s *Z*) for homework, indicating that this variable might indeed enhance school effectiveness. However, recent studies have provided evidence that homework assignments are not per se performance-enhancing. For instance, the effectiveness of homework seems to depend on the quality of the tasks assigned. Homework assignments that are perceived to be well selected and cognitively challenging are positively associated with students’ achievement (Dettmers et al., 2010).

A further potential predictor of the effectiveness of homework assignments is parental homework involvement. Parental involvement in homework completion is commonly expected by schools, teachers, and parents (Patall et al., 2008), all of whom believe that parental homework involvement is vital for students’ school performance (Epstein, 1986; Trautwein et al., 2009). Thus, numerous guidelines for parents exist, aiming to improve parents’ abilities to successfully support homework completion (e.g., U.S. Department of Education, 2005). In the US, more than 80% of parents believe that homework is important for learning. Even though 51% of parents reported that students should do their homework on their own, on average, 73% of parents reported helping their child with homework completion. However, at the same time, 29% of parents perceived a negative impact of homework on family life (Markow et al., 2007). Given this high percentage of parents who become involved in their children’s homework completion and a substantial number of parents who complained about family stress due to homework, the question arises concerning whether and under which conditions parental homework involvement is beneficial. Parental homework involvement is one facet of parental involvement in schooling, which is believed to be one of the key promoters of students’ school-related outcomes such as achievement, motivation, and well-being (e.g., Fan and Chen, 2001; Epstein, 2005; Hill and Tyson, 2009; Ma et al., 2016). The importance attached to parental behavior in their children’s education becomes apparent in the development of significant educational policies [e.g., U.S. Department of Education, 2002] and projects fostering educational partnerships [e.g., teachers involve parents in schoolwork (TIPS, Van Voorhis, 2003), and teachers involving parents (TIP, Hoover-Dempsey et al., 2002)], which stresses the role that parents play in their children’s education. Indeed, meta-analyses have provided evidence that regardless of their socioeconomic background and race, students’ school achievement can be improved if their parents become involved in their education (e.g., Fan and Chen, 2001; Hill and Tyson, 2009; Ma et al., 2016). However, parental involvement represents a multifaceted behavior that can take place in school (school-based involvement: e.g., community services at school) or at home (home-based involvement; Grolnick and Slowiaczek, 1994, Hoover-Dempsey and Sandler, 1997). Previous studies analyzing the effectiveness of parental homework involvement have demonstrated mixed results about the link between this type of involvement and students’ school performance, with some studies having found a positive link (e.g., Van Voorhis, 2003; Xu, 2004; Silinskas and Kikas, 2011) while others have found a negative link (e.g., Xu et al., 2010; Dumont et al., 2012). These studies have suggested that one should consider how homework involvement is assessed. Most importantly, it is the quality (and not the amount) of homework involvement that is crucial for student outcomes (e.g., Knollmann and Wild, 2007a,b; Dumont et al., 2014; Gonida and Cortina, 2014; Moroni et al., 2015).

The present study was built upon these previous studies, aiming to shed light on factors that might improve the quality of parental homework involvement and thereby student outcomes (achievement and students’ well-being). In recent years, the concept of FSP has become well known, as it is believed to foster parental abilities to help their children with learning. Studies have proven that a positive contact between schools and parents is related with higher parental school involvement (Ames et al., 1993; Kohl et al., 2000; Patrikakou and Weissberg, 2000). The aim of the present study was threefold. Our first research question concerned the relationship between the quality of parental homework involvement and four student outcomes: achievement in mathematics and reading as well as well-being at home and school. Second, we analyzed the association between effective family-school communication (EFSC) on the one hand and parental homework involvement and the four student outcomes on the other hand. Third, we investigated the interplay between our variables, namely whether parental homework involvement mediates the association between EFSC and the four student outcomes.

## Predictors and Outcomes of Parental Homework Involvement

Past research has suggested that parental homework involvement is a multidimensional construct including two distinct types of help: quantitative help (e.g., doing homework with the child, providing answers) and qualitative help (e.g., avoiding distractions, providing rules for homework completion, providing support for finding answers) (e.g., Gonida and Cortina, 2014). Although the general term of parental involvement is accepted to be one of the key promoters of learning, parental homework involvement is not always positively related with desired school outcomes such as achievement. For example, Xu et al. (2010) found the frequency of parental homework help to be negatively related with student reading achievement and raised the question of how parents should help with homework. The authors concluded that parents should provide a suitable learning environment for homework completion to foster self-regulated learning and children’s autonomy. Moroni et al. (2015) operationalized parental involvement as a multidimensional construct in terms of quantity and quality and examined how the quantity and different qualities of homework involvement were associated with student achievement. Controlling for prior achievement and parental socioeconomic background, they found the frequency of help to be negatively associated with the development of student achievement. However, in terms of homework quality, the authors found opposing effects depending on how homework quality was operationalized. While supportive homework help had positive effects on students’ achievement, intrusive homework help was negatively related with later achievement. Dumont et al. (2014) analyzed longitudinal data of 2,830 student-parent dyads (grades 5 and 7) who reported about the quality of parental homework involvement, their socioeconomic background, and desired student outcomes (e.g., reading achievement, reading effort). Adopting the perspective of self-determination theory (SDT, Deci and Ryan, 1987, 2000), parental homework involvement was conceptualized by three dimensions: parental control, parental responsiveness, and parental provision of structure. The analyses revealed a reciprocal relationship between parental homework involvement and student outcomes. Low achievement in grade 5 predicted higher later parental homework control in grade 7, while high parental control in grade 5 was related with lower achievement in grade 7. A positive reciprocal relationship was found for parental involvement in terms of structure and responsiveness on the one hand and desired student outcomes – such as high achievement – on the other hand. Types of parental involvement did not depend on parental socioeconomic background.

Supportive parental homework involvement – such as the parental provision of autonomy support or structure – is not only positively associated with students’ academic performance, but it is also believed to be beneficial for students’ well-being (e.g., Hoover-Dempsey et al., 2002; Pekrun et al., 2002). It is assumed that supportive parental behavior fulfills students’ basic needs proposed by SDT, namely the need for autonomy, relatedness, and competence (Grolnick, 2009). Basic needs satisfaction may result in an internalization of uninteresting and boring activities such as doing homework into personally important activities, thereby fostering performance and well-being (Deci and Ryan, 2000). To date, few studies have provided evidence of this linkage. Knollmann and Wild (2007b) conducted a survey with 181 German students concerning their parents’ provision of autonomy support, emotional support, and support for competence during parental instruction at home. The authors found autonomy and emotional support to be positively associated with joy. By contrast, lower levels of autonomy and emotional support predicted higher rates of students’ anger. Moreover, according to Kenney-Benson and Pomerantz (2005), greater autonomy-supportive homework help of mothers was found to be associated with less depressive symptoms compared to controlling mothers.

To sum up, the quality of parental homework help seems to be related with differences in students’ well-being and academic achievement. In line with the assumptions of SDT, numerous studies suggest that autonomy- and competence-supportive parental homework involvement may increase students’ experiences of autonomous and competent learning experiences, which in turn fosters desired (learning) outcomes. Hence, the question arises about factors that may influence the quality of parental homework involvement. Gonida and Cortina (2014) investigated predictors and consequences of parental homework involvement. The authors asked Greek parents to rate different types of parental homework involvement (autonomy-supportive homework involvement, controlling homework involvement, and interference). Moreover, parents and their children provided information on achievement goals, academic efficacy, and school grades. Structural equation models revealed that autonomy-supportive homework involvement was predicted by parent mastery goals while parent performance goals predicted controlling homework involvement. Moreover, the authors provided evidence that parental beliefs for children’s self-efficacy were negatively associated with parent control and interference, but positively related with parent encouragement for cognitive engagement as supplementary to homework. Furthermore, this study demonstrated that low parent beliefs in their children’s abilities to complete homework successfully may result in an inappropriate way of homework involvement in terms of control and interference.

However, to our knowledge, little is known about further factors that might promote the quality of parental homework involvement. Given the important role of parents in their children’s education, the present study addressed this research deficit and aims to shed light on potential predictors of parental homework involvement. Students and their parents spend a lot of time with homework, although parents report barriers to their homework involvement in the sense that – for instance – they sometimes feel unable to provide appropriate help and they tend to require recommendations from teachers about how to help with homework (Kay et al., 1994). In the present study, we assume EFSC to be a potential predictor of the quality of parental homework involvement. A welcoming school climate and recommendations for homework involvement might act as an invitation to involve as they indicate that parental involvement is desired and important (Becker and Epstein, 1982; Epstein, 1986; Epstein and Van Voorhis, 2001). In the next section, we present a theoretical model of parental involvement in schooling and corresponding empirical studies.

## Defining Parental Involvement in Schooling

Parental involvement in schooling is seen as a key strategy to improve students’ success in school. Indeed, a strong body of evidence suggests that parental involvement in schooling is positively associated with various desired school-related outcomes such as school performance and positive affect (e.g., Fan and Chen, 2001; Hill and Tyson, 2009; Ma et al., 2016). According to Epstein (1995), supportive and event-independent communication between parents, school principals, and teachers may result in a deepened mutual understanding about school as well as improved support of students by their parents and teachers. Hoover-Dempsey and Sandler (1995, 1997, 2005) developed a theoretical model of parental involvement process that describes the antecedents and consequences of parental involvement in schooling. The model proposes five sequential levels to explain factors that might influence parents’ choice to become involved, their resulting forms of involvement and their consequences. The *first level* identifies three reasons for parents to become involved in their children’s schooling: parents’ perceived role construction (e.g., whether they feel obliged to help), their perceived invitations to involvement from the school, the teacher, and their child, as well as their sense of efficacy for helping their children. *The second level* suggests two forms of parental involvement, namely home- and school-based involvement, both of which include encouragement, modeling, reinforcement, and instruction. At the *third level*, children’s perceptions of the four types of parental involvement (encouragement, modeling, reinforcement, and instruction) are described. The *fourth level* describes mediating variables, namely child attributes and use of developmentally appropriate parental involvement. Finally, the *fifth level* focuses on school achievement (for a more detailed description, see Hoover-Dempsey et al., 2005; Hoover-Dempsey and Sandler, 2005). The focus of the present study was on the first level of the model, which deals with the question of why parents become involved in their children’s schooling. Hoover-Dempsey and Sandler’s model identifies three sources of invitations for parents to become involved in schooling: invitations from the school, the child, and the child’s teachers. Invitations from the school might include a welcoming school climate and the perception that parental involvement is crucial and desired in supporting children’s learning and achievement. Teachers can foster parental involvement through direct requests for involvement in children’s education; for instance, by encouraging parents to talk about school activities with their child. Finally, children’s attributes (e.g., prior achievement in school) might act as an invitation to become involved. Numerous previous studies have provided evidence regarding the relationship between level 1 variables (reasons for becoming involved) and the amount of involvement in school and at home (e.g., Green et al., 2007). For example, Green and colleagues used the data of 853 parents of elementary and middle school students to examine associations between antecedent factors (level 1) and different forms of parental involvement (level 2) proposed in the theoretical model by Hoover-Dempsey and Sandler. Regression analyses revealed that parental self-efficacy, child invitations, and parents’ time and energy were positively associated with the amount of home- and school-based involvement. Moreover, teacher invitations predicted the quantity of parents’ school-based involvement. Yotyodying and Wild (2014) examined whether parental perceptions of invitations for involvement from the school and teachers in a German and Thai sample as one among other predictors variables would predict two distinct forms of home-based parental involvement: authoritative (greater autonomy support and responsiveness) and authoritarian (greater control and structure). In the German sample, the significant results showed that parental perceptions of invitations from the school and teachers were negatively associated with both authoritative and authoritarian ways of involvement. This means that parents who prefer either authoritative or authoritarian ways of involvement tend to neglect becoming involved if they feel less invited by the school and teachers.

However, it should be critically noted that Hoover-Dempsey and Sandler’s model as well as most related empirical studies have focused particularly on the quantity (how often parents become involved) of parental involvement, while the quality (the ways in which parents become involved) of parental involvement has been neglected in many studies.

The present study aims to expand the existing body of knowledge by taking the quality (instead of the quantity) of parental involvement into account. In order to gain deeper insights into the mechanisms of parental involvement, we concentrated on one subdimension of parental involvement in schooling: parental homework involvement. Adopting a self-determination perspective on parental need support, the quality of parental homework involvement was differentiated into two dimensions of parental supportive behavior: autonomy support and competence support. The following research questions arise from the above explanations: is high-quality parental homework involvement positively associated with students’ achievement and well-being? Moreover, how can high-quality parental involvement be fostered?

## Family-School Partnerships in Germany

Given the importance of improving parental involvement, scholars have attempted to identify variables that increase beneficial parental involvement. In recent years, the concept of family-school partnerships (FSPs) has become well known as an instrument that might foster parental choice to become involved in their children’s education and parental abilities to help their children with learning. Indeed, studies have proven that successful FSPs are positively associated with students’ performance (see Henderson and Mapp, 2002; Sheldon, 2003). A positive contact between teachers and parents increases the probability that parents become involved in their children’s education (Ames et al., 1993; Kohl et al., 2000; Hoover-Dempsey and Walker, 2002). Moreover, information from teachers about classroom learning and instruction shape parental strategies to become involved (Ames et al., 1993). In order to strengthen successful FSP, in 1997, the National Parent Teacher Association (PTA) published the National Standards for Family-School Partnership for the US context. These standards build upon Epstein’s typology of parental involvement (see Epstein, 2001) and provide a practical guideline to implement FSP. The PTA proposed six standards: (1) welcoming all families into the school community, (2) communicating effectively, (3) supporting student success, (4) speaking up for every child, (5) sharing power, and (6) collaborating with community (for more information, see Parent-Teacher Association, 2009). Compared to the US, to our knowledge, in Germany, much less is known about the concept and the benefits of well-functioning FSP (Wild and Yotyodying, 2012). To date, contacts between schools and parents are rare and not very effective and mostly take place at parent evening events (Wild and Hofer, 2002; Sacher, 2008). Moreover, conversations between teachers and parents mainly concern learning problems and students’ grades (Wild and Lorenz, 2010; Wild and Yodyodying, 2012). For this reason, the Vodafone Foundation in collaboration with a scientific expert committee (see Sacher et al., 2013) recently proposed a compass for family-school partnerships for the German context comprising four different standards. The development of the four indicators is based on the six PTA standards described above, although the standards were adapted to the German context and the sixth standard “collaborating with community” was excluded for Germany. Standard A “Welcoming and Meeting Culture” describes a welcoming and friendly school climate that can be characterized by mutual respect and the inclusion of all stakeholders. Standard B “Various and Respectful Communication” is characterized by a regular and routine information exchange between the school, teachers, and parents, the use of various ways of information, and a regular information exchange between all stakeholders. Standard C “Educational Cooperation” focuses on parental participation in school life, the encouragement of parents to support their children with learning, the information about external school-related offers, and it emphasizes the role of parents as interceders of their child. Finally, Standard D “Parent Participation” describes the provision of information about parents’ participatory rights, the possibility for parents to participate in school decisions, and the inclusion of social, political, and external networks in school life. To our knowledge, little is known about whether the proposed standards would be met in German schools and whether they would help to ensure parental involvement, especially parental help with homework. For this reason, we developed and validated a parental questionnaire to assess parental perceptions on different aspects of FSP based on the proposals of Vodafone’s scientific committee.

The aim of the present study was to identify factors that might promote the quality of parental homework involvement. In consideration of Hoover-Dempsey and Sandler’s model, which identifies three reasons for parents to become involved (their role construction, their perceived invitations, and their sense of competence to help) and previous studies (e.g., Becker and Epstein, 1982; Epstein, 1986; Epstein and Van Voorhis, 2001), we proposed that EFSC would foster the quality of parental homework involvement. In order to operationally characterize EFSC, we relied on three indicators of Standard B “Various and Respectful Communication” and developed three scales (15 items) assessing EFSC. B1 “Information Exchange” describes a regular and routine information exchange between the school, teachers, and parents. Standard B2 “Various Forms of Communication” focuses on the use of the variety of ways of communication between the school and parents (e.g., email, homepage, etc.). B3 “School Transitions” refers to a regular knowledge transfer and information exchange between schools, teachers, and parents during school transitions.

## The Present Study

The present study addresses three research deficits. *First*, parental school involvement is a multidimensional construct comprising both parental involvement at school and parental involvement at home. Research findings on parental school-based involvement are not transferable to home-based involvement, given that the context of the two forms of involvement differs. The present study concentrates on home-based involvement, more precisely on homework involvement as one facet of it. Research on parental homework involvement has provided evidence for the need to distinguish between the quality and quantity of parental involvement, whereby it is the quality (rather than the quantity) of involvement that matters for desired student outcomes (e.g., Dumont et al., 2014; Moroni et al., 2015). Adopting a self-determination perspective on parental need support, the quality of parental homework involvement was differentiated into two dimensions of parental *supportive* behavior: autonomy support and competence support. Our first research question concerned the relationship between parental homework involvement and four different student outcomes: well-being at school, well-being at home, mathematics achievement, and language achievement. *Second*, the concept of FSP is well known and has been much studied in the US context. There is clear consensus that parental involvement in schooling is beneficial and that a successful implementation of FSP fosters parental involvement, thereby promoting student achievement (Ames et al., 1993; Kohl et al., 2000; Fan and Chen, 2001; Henderson and Mapp, 2002; Hoover-Dempsey and Walker, 2002; Sheldon, 2003; Epstein, 2005; Hill and Tyson, 2009; Ma et al., 2016). However, theoretical models and much FSP research have concentrated on the effects of FSP on the quantity (the amount) of involvement, while the relationship between FSP and the quality of parental school involvement and student outcomes remains unclear. Moreover, to our knowledge, in Germany, much less is known about effects of the implementation of successful FSP. The four standards of FSP proposed by the Vodafone Foundation and a scientific expert committee (Sacher et al., 2013) are the first theoretical compass for FSP in the German context. To date, the concept has not been empirically analyzed in Germany and it is unclear whether a successful implementation of FSP is related to parental school- and home-based involvement. Our second research question thus concerned the relationship between EFSC (as one facet of FSP) and parental homework involvement and the different student outcomes. Finally, our *third* research question focuses on the mediating role of parental homework involvement for the relationship between EFSC and the four student outcomes. In order to investigate these relationships, we assumed that socioeconomic status and student gender may act as barriers to parental homework involvement (e.g., Hornby and Lafaele, 2011). Thus, there is a need to control for both variables.

## Materials and Methods

### Data Source and Sample

Between winter 2015 and spring 2018, we conducted an online survey with parents of primary and secondary school students. The sample included 309 parents (82% mothers; *M* age = 42 years) of school students. Of the participants’ children (*M* age = 12 years, SD = 3.58), 55% were girls and 44% attended elementary schools. Parents were asked to rate the amount of EFSC and their homework support. Moreover, parents rated children’s well-being and school achievement. The percentage of missing data was low for the variables analyzed here (on average 0.91%).

### Instruments

#### Effective Family-School Communication

EFSC was assessed with three indicators of Standard B “Various and Respectful Communication” and comprises: (1) “Regular and event-independent information exchange” [five items, e.g., “If I am (or my child is) concerned about something, I can discuss this with the teachers, the school principal, or other parents.”], (2) “various forms of communication” [six items, e.g., “The school communicates with parents in different ways (e.g., email, telephone, and website).”], and (3) “school transitions” [five items, e.g., “The school management and teachers actively inform parents and children about the possibilities when making their school decisions.”]. All items were rated on a 4-point Likert scale ranging from 1 = “strongly disagree” to 4 = “strongly agree.” Cronbach’s alpha for EFSC was 0.91. The psychometric properties of the subscales are shown in Table 1.

**Table 1:** Means, standard deviations, and internal consistencies for all study variables.

Study variables	*M*	SD	*α*
B1: Information exchange	2.87	0.57	0.74
B2: Various forms of communication	2.90	0.69	0.86
B3: School transitions	2.94	0.68	0.78
Autonomy-supportive homework involvement	3.30	0.55	0.74
Competence-supportive homework involvement	3.51	0.58	0.77
Mathematics achievement	3.27	0.73	0.95
Language achievement	3.34	0.67	0.92
Well-being school	7.60	0.91	
Well-being at home	8.70	0.49	

#### Parental Homework Involvement

Adopting a self-determination perspective on parental need support, the quality of parental homework involvement was differentiated into two dimensions of parental supportive behavior (Katz et al., 2011): (1) *autonomy-supportive homework involvement* was assessed with five items (e.g., “While working on homework, I am willing to hear my child provide answers that are different from mine.”); and (2) *competence-supportive homework involvement* comprised three items (e.g., “I am glad if my child provides an answer in homework that is different from what is expected but is interesting.”). Items were rated on a 4-point Likert scale ranging from 1 = “strongly disagree” to 4 = “strongly agree.” Cronbach’s alpha for parental homework support was 0.83.

#### Well-Being

In the present study, we differentiated between student well-being at home and in school. Using two different 10-point ladders (Cantril, 1965) ranging from 1 *(they are doing really poorly in school/at home*) to 10 (*they are doing really well in school/at home*), parents were asked to rate how their children feel about their lives in school (well-being at school) and at home (well-being at home).

#### School Achievement

School achievement was assessed with two indicators. Parents were asked to rate their children’s *mathematics achievement* in mathematics with three items on a 4-point Likert scale: (a) my child is (1) *not good*...(4) *very good* in arithmetic, (b) my child makes (1) *many mistakes*...(4) *very few mistakes* in arithmetic, (c) arithmetic is (1) *difficult*...(4) *easy for my child*. Cronbach’s alpha of this scale was 0.95. *Language achievement* comprised six items about the reading and writing abilities of their children. Parents were asked to judge the items on a 4-point Likert scale, e.g., (a) my child makes (1) *so many mistakes*...(4) very few mistakes when reading, (b) writing is (1) *difficult*...(4) *easy for my child*. Cronbach’s alpha of this scale was 0.92.

#### Socioeconomic Status

Parental socioeconomic status (SES) was assessed using the CASMIN classification (Comparative Analysis of Social Mobility in Industrial Nations; König et al., 1988), a comparative educational scale. Parents provided information on their school education (e.g., A-level) and their professional education (e.g., university degree). In order to build a CASMIN index, both variables of each parent were combined and then distinguished into three different educational levels (elementary, intermediate, and higher level). According to this classification, 2% of the parents reported having a SES at the elementary level, 15% at the intermediate level, and 83% at the higher level. We created a dummy variable for the SES, coded as 1 if participants reported a CASMIN at the higher level, and 0 if participants reported a lower CASMIN.

### Statistical Analyses

In order to test our hypotheses empirically, structural equation modeling (SEM) analyses were performed. SEM allows testing the relationships postulated in the present study. All analyses were performed using MPlus 7.4 (Muthén and Muthén, 2012–2014). EFSC was operationalized as a latent construct, measured by three manifest indicators (regular and event-independent information exchange, various forms of communication, and school transitions). Parental homework involvement was measured by two indicators: autonomy- and competence-supportive homework involvement. In order to control for parental SES and student gender, we estimated the links between both variables and the mediator (parental homework involvement), as well as the outcomes (achievement and well-being). Standardized parameter estimates of models with good fit were reported. Model fit was evaluated by considering the *χ*^2^ test, the comparative fit index (CFI), the Tucker Lewis Index (TLI), the standardized root mean square residual SRMR, and the root mean square error of approximation RMSEA. According to Schreiber et al. (2006), a nonsignificant *χ*^2^ test, and a value of 0.95 or higher for the GFI and CFI indicates an acceptable model fit. The average percentage of missing data ranged from 0 to 3.2%. Since the proportion of missing values was low and could be assumed to be missing at random (MAR), it was dealt with the full information maximum likelihood estimation (FIML) implemented in MPlus. In FIML, all information available is considered to estimate the parameters. FIML produces unbiased parameter estimates and standard errors and is superior to traditional deletion methods (e. g., listwise and pairwise deletion) (Schafer and Graham, 2002).

## Results

### Descriptive Statistics and Zero-Order Correlations

Table 1 presents means, standard deviations, and Cronbach’s alpha for the study variables. Parents’ average ratings of EFSC were moderately above the scale midpoint, indicating a rather frequent contact between schools and parents and a “well-functioning information flow.” Parents report a regular and routine information exchange between the school, teachers, and parents. Moreover, as perceived by parents, most schools used various forms to communicate with parents, e.g., email, homepage, etc. Finally, parents perceived a regular knowledge transfer and information exchange between schools, teachers, and parents during school transitions. Parental ratings of homework support were significantly above the scale midpoint. Hence, from a self-determination perspective on parental need support, parents reported a rather high quality of parental homework involvement. They reported being autonomy- and competence-supportive during homework completion. Achievement was rated on a 4-point Likert scale. As shown in Table 1, on average, parents rated their children’s achievement in mathematics and reading high. While well-being was also rated high. On a 10-point ladder with high values indicating high well-being, parents perceived their children to feel rather well in school and very well at home.

In order to gain insights into the association between the research variables, Table 2 presents the Pearson’s correlation coefficients between all analyzed variables. The significant correlations ranged from *r* = 0.14 (*p* < 0.05) to *r* = 0.53 (*p* < 0.01). As expected, EFSC was positively associated with supportive parental homework involvement (*r* = 0.39, *p* < 0.01), indicating that a well-functioning contact and information flow between schools, teachers, and parents is related with autonomy- and competence-supportive parental homework behavior. Moreover, high values in EFSC were related with well-being at school (*r* = 0.35, *p* < 0.01) and home (*r* = 0.14, *p* < 0.05). Finally, EFSC was positively associated with achievement in mathematics (*r* = 0.20, *p* < 0.01) and language (*r* = 0.20, *p* < 0.01). The same holds for autonomy- and competence-supportive parental homework behavior. The variable was positively related with well-being at school (*r* = 0.16, *p* < 0.01) and home (*r* = 0.42, *p* < 0.01) and with school achievement (mathematics: *r* = 0.24, *p* < 0.01; language: *r* = 0.47, *p* < 0.01). In sum, the intercorrelations revealed that our research variables are related to each other in the expected way. In order to draw further conclusions about their relationship and answer our research questions, we estimated regression analyses and a structural equation model to predict parental homework involvement, school achievement, and well-being, as well as to test the mediating role of parental homework involvement for the potential association between EFSC and our outcome variables.

**Table 2:** Intercorrelations among study variables.

			Well-being		Achievement		
	**EFSC**	**Support**	**At school**	**At home**	**Math**	**Language**	**Female**
EFSC								
Parental homework involvement	0.39**			
Well-being at school	0.35**	0.16**			
Well-being at home	0.14*	0.42**	0.53**			
Mathematics achievement	0.20**	0.24**	0.26**	0.25**	
Language achievement	0.20**	0.47**	0.25**	0.30**	0.35**	
Female	0.03	0.01	0.08	−0.04	−0.03	0.01	

Note: EFSC = Effective family-school communication, N = 309, *p < 0.05, **p < 0.01

### The Relationship Between Parental Homework Involvement and Student Outcomes

In the first step, we performed a regression analyses to predict students’ well-being at school and home and their achievement in mathematics and language. The results are shown in Table 3, model 1. Model fit was rated based on the *χ*^2^ test, the CFI, the TLI, the SRMR, and the RMSEA. The model revealed good model fit to the data, *χ*^2^ (522, *N* = 309) = 5.03, CFI = 1.00, TLI = 1.00; SRMR = 0.01, RMSEA = 0.01. As can be seen in Table 3, controlling for socioeconomic status and gender (female), parental homework involvement predicted well-being at school (*β* = 0.15, *p* < 0.05), well-being at home (*β* = 0.42, *p* < 0.01), mathematics achievement (*β* = 0.24, *p* < 0.01), and language achievement (*β* = 0.46, *p* < 0.01). Hence, according to their parents, students whose parents are autonomy- and competence-supportive during homework completion feel more well at school and home and achieve better results in mathematics and language compared to other students. The variance explained was between 3% (for well-being at school) and 23% (for language achievement).

**Table 3:** Associations among effective family-school communication, parental homework involvement, well-being at school, well-being at home, mathematics achievement, and language achievement after controlling for child gender and parental SES.

			Well-being at school		Well-being at home		Mathematics achievement		Language achievement	
Model 1			*β*	SE	*β*	SE	*β*	SE	*β*	SE
Parental homework involvement			0.15*	0.06	0.42***	0.06	0.24***	0.06	0.46***	0.05
Female			0.08	0.06	−0.08	0.05	−0.10	0.06	0.11*	0.05
SES			0.05	0.06	0.10*	0.05	0.05	0.06	−0.01	0.05
*R*^2^			0.03		0.19***		0.07*		0.23***	
	**Parental homework involvement**		**Well-being at school**		**Well-being at home**		**Mathematics achievement**		**Language achievement**	
**Model 2**	***β***	**SE**	***β***	**SE**	***β***	**SE**	***β***	**SE**	***β***	**SE**
Effective family-school communication	0.40***	0.06	0.34***	0.06	0.16**	0.06	0.22***	0.06	0.19**	0.06
Female	0.00	0.06	0.05	0.05	−0.08	0.06	0.08	0.06	0.12*	0.06
SES	0.04	0.06	0.10	0.05	0.12*	0.06	−0.12*	0.06	0.01	0.06
*R*^2^	0.16**		0.14**		0.04		0.06*		0.06*	

Note: N = 309, *p < 0.05, **p < 0.01, ***p < 0.01

### The Relationship Between Effective Family-School Communication and Parental Homework Behavior and Student Outcomes

The next section presents the findings of regression analyses to empirically test the assumed relationships between EFSC and the other variables of this study. Table 3, model 2, shows the results for the prediction of parental homework involvement, well-being at school and home, as well as achievement in mathematics and language. The model revealed good model fit to the data, *χ*^2^ (22, *N* = 309) = 32.21, CFI = 0.99, TLI = 0.97; SRMR = 0.02, RMSEA = 0.04. As can be seen in Table 3, after controlling for socioeconomic status (CASMIN) and gender (female), regression analysis indicated that EFSC predicts parental homework support (*β* = 0.40, *p* < 0.01). Thus, parents whose children visit schools with a well-functioning EFSC reported being more autonomy- and competence-supportive during homework completion. The variance explained was 16% for this model.

The next two columns show the results for the prediction of students’ well-being. After controlling for socioeconomic status and gender, the results revealed a positive relationship between parental homework support and well-being at school (*β* = 0.34, *p* < 0.01), as well as well-being at home (*β* = 0.16, *p* < 0.01). Hence, the results indicate that children whose parents perceive themselves as being autonomy- and competence-supportive during their children’s homework completion feel more well at school and home compared to other children. The variance explained was 14% for well-being at school and 4% for well-being at home. The last two columns in Table 3 present the results for the prediction of mathematics and language achievement. Mathematics achievement was predicted by EFSC (*β* = 0.22, *p* < 0.01) and female gender (*β* = −0.12, *p* < 0.05). Language achievement was predicted by EFSC (*β* = 0.19, *p* < 0.05) and female gender (*β* = 0.12, *p* < 0.05). The results thus indicate that a well-functioning communication between schools, teachers, and parents may improve students’ achievement in mathematics and the language domain. The percentage of variance explained was 6% for mathematics achievement and 6% for language achievement. In sum, the study provided first evidence for the German context that EFSC may improve the quality of parental homework support in terms of autonomy and competence support. Moreover, EFSC proved to be beneficial for students’ well-being at home and may foster mathematics and language achievement.

### Mediating Role of Parental Homework Help

**Figure 1:**
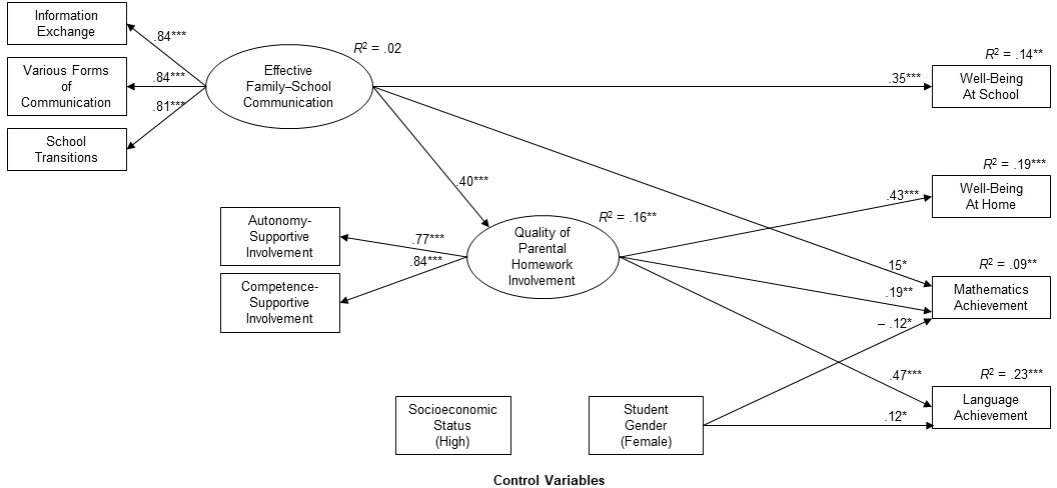
Structural model for the associations between effective family-school communication, quality of parental homework involvement, and students’ desired outcomes after controlling for parental SES and student gender. Note: *N* = 309, *p < 0.05, **p < 0.01, ***p < 0.001. For reasons of simplification, only significant path coefficients are shown.

In order to gain deeper insights into the mechanisms of the relationships found in the previous section, our third research question concerned the mediating role of parental homework involvement in the relationship between EFSC and well-being as well as school achievement. Figure 1 shows the results of a structural equation model. For the sake of easier readability, only significant pathways are shown. Overall, the model shows excellent model fit to the data: *χ*^2^ (22, *N* = 309) = 32.21, CFI = 0.99, TLI = 0.97; SRMR = 0.02, RMSEA = 0.04.

After controlling for socioeconomic status and female gender, EFSC was found to be positively associated with parental homework involvement (*β* = 0.40, *p* < 0.001). Compared with the regression coefficients found in regression analyses (see Table 3, model 2), the relationship between EFSC and well-being at school remained at a substantial level (*β* = 0.35, *p* < 0.001). However, the coefficient for the relationship between EFSC and mathematics achievement slightly decreased from *β* = 0.19 to *β* = 0.15 (*p* < 0.05). Moreover, the inclusion of parental homework involvement in our analyses led to reduced coefficients for the relationship between EFSC and well-being at home (*β* = −0.01) and language achievement (*β* = 0.00). These relationships were no longer statistically significant.

In addition to the direct effects, indirect effects of the predictor EFSC on well-being and achievement as mediated by parental homework support were examined. The inclusion of the mediator variables partly led to different regression coefficients for EFSC, indicating the mediating role of parental homework involvement. The indirect effect of EFSC on well-being at home was statistically significant (*β* = 0.17, *p* < 0.01), indicating a full mediation of the relationship. The indirect relationship between EFSC and mathematics achievement was statistically significant (*β* = 0.07, *p* < 0.01), indicating a partial mediation. Furthermore, the indirect effect of EFSC on language achievement was statistically significant (*β* = 0.19, *p* < 0.001), indicating a full mediation. Because the link between parental homework involvement and well-being at school was not found, the indirect effect was not examined.

Together, the results demonstrated that the quality of parental homework support fully mediated the relations of EFSC with well-being at home and language achievement, while it partially mediated the relations of EFSC with mathematics achievement. Hence, EFSC had significant positive indirect effects on well-being at home and student’s achievement.

## Discussion

The primary aim of the present study was to analyze predictors and consequences of high-quality parental homework involvement. More precisely, we tested whether EFSC would predict the quality of parental homework involvement and in turn students’ well-being and school achievement. The participants of the study were 309 parents of primary and secondary school students in Germany who participated in an online survey. Three research questions were addressed. Our first research question addressed the role of parental homework involvement. With respect to the SDT, parental homework involvement was operationalized as autonomy- and competence-supportive. Based on regression analyses, we tested the relationship between parental homework involvement and four different student outcomes: well-being at school, well-being at home, mathematics achievement, and language achievement. Our second research question focused on the associations among EFSC, the quality parental homework involvement, students’ well-being, and school achievement in two domains. Our third research question concerned the mediating role of parental homework involvement for the relationship between EFSC and the four student outcomes.

In line with our assumptions made for the first research question, we found high-quality parental homework involvement to be positively associated with students’ well-being at school and at home, as well as with students’ achievement in mathematics and language. This result supports the results of earlier studies concluding that the effectiveness of parental homework involvement depends on its quality (e.g., Knollmann and Wild, 2007a,b; Dumont et al., 2014; Gonida and Cortina, 2014; Moroni et al., 2015).

Past research has suggested that (the quantity of) parental involvement in schooling is beneficial for different student outcomes (e.g., Fan and Chen, 2001; Hill and Tyson, 2009; Ma et al., 2016). Building upon Hoover-Dempsey and Sandler’s model of parental involvement process (Hoover-Dempsey and Sandler, 1995, 1997, 2005) and recent studies (e.g., Green et al., 2007), we assumed an EFSC to be positively associated with parental homework involvement and different student outcomes. Using a recently developed instrument to assess parental perceptions of EFSC, our second research question focused on the relationship between EFSC and parental homework involvement and the four student outcomes. Our results of regression analyses provided evidence for the predictive power of EFSC for the quality of parental homework involvement and all four different student outcomes. As previously mentioned, Hoover-Dempsey and Sandler’s model underlines specific invitations from school (teachers’ attempt to invite parents to become involved) as one of crucial predictors of the quantity of parental involvement. Our results added to this model in the sense that EFSC – which might function as a reason to become involved – predicts the quality of parental involvement in schooling. Our study extends previous research on the model as it considers the need to distinguish between the quantity and quality of involvement. To our knowledge, our study is the first to provide evidence of the predictive power of EFSC for high-quality parental homework involvement. Contrary to our results, Yotyodying and Wild (2014) found teacher invitations to be related with the amount of parental home-based involvement but not with differences in the quality of home-based involvement. The authors concluded that teachers presumably increase parents’ awareness of the importance to become involved in schooling, but that they possibly do not provide information about how parents might help their children in school-related topics. In their study, the authors asked parents to rate the extent to which they perceive that their school involvement is expected and requested. In the present study, parents were asked to rate an EFSC in a way that a regular and event-independent information exchange exists, that the schools and teachers use various forms of communication and that information about school transitions is provided. An EFSC might not only act as an invitation to help but it also possibly provides parents with information concerning how to help their children in school-related topics. In addition, our results indicated that EFSC positively contributed to all four student outcomes. These results were also in line with previous studies finding that successful FSPs help to improve students’ performance (e.g., Henderson and Mapp, 2002; Sheldon, 2003).

In order to address our third research question, we examined the mediating role of the quality of parental homework involvement. Controlling for socioeconomic status and students’ gender, SEM analyses showed that the associations between EFSC and three of the four student outcome variables (well-being at home, mathematics achievement, and language achievement) were (partially) mediated by the quality of parental homework involvement. The results of the present study thus highlight the role of EFSC as a key performance factor that helps to improve the quality of parental homework involvement, thereby promoting student outcomes. In addition, our findings on the crucial mediating role of parental homework involvement in the associations between EFSC and well-being at home and school achievement were in line with the assumptions of self-determination theory (SDT: Deci and Ryan, 1987, 2000). Accordingly, the parental provision of autonomy and competence support tend to satisfy the basic needs of their children (autonomy and competence), and in turn it might thus result in improved well-being. Indeed, earlier studies (Chirkov and Ryan, 2001; Niemiec et al., 2006; Yotyodying, 2012) have provided evidence for the relationship between parental autonomy support and well-being (e.g., life satisfaction, positive affect, school satisfaction, positive academic emotions). Our results suggest that an EFSC results in a higher quality of parental homework involvement (in terms of autonomy and competence support), which in turn leads to increased well-being at home compared to other children. Concerning achievement, our results were in line with previous studies providing evidence of a positive relationship between parental involvement in schooling and students’ achievement (e.g., Fan and Chen, 2001; Hill and Tyson, 2009; Ma et al., 2016), although they extend these studies by showing the mediating role of parental homework involvement for this relationship. Hence, EFSC results in high-quality parental homework involvement and is in turn related to achievement.

### Practical and Scientific Implications of the Study

Recent studies have shown that strong family-school partnerships (FSPs) may help to improve parental involvement. From a scientific view, the findings of the present study supplement this research in two aspects: first, to our best knowledge, to date only little is known about the relationship between FSP and parental homework involvement. We were able to confirm that EFSC (as an indicator of FSP) may help to improve the quality of parental involvement at home, which in turn supports well-being and school achievement of students. Second, compared to the US, in Germany, much less is known about the benefits of FSP (Wild and Yotyodying, 2012). We have been able to show that German parents evaluate the communication between families and schools positively. However, according to Hoover-Dempsey and Walker (2002), various barriers might hinder well-functioning FSP such as parents having a low level of education, inflexible working hours, or low language skills. For schools, structural elements such as personnel resources influence FSP. Hence, our results of the present study hold strong importance for different groups. Administrators may use our results to implement teacher and parent training programs aiming to promote the awareness of teachers and parents about the consequences of parental involvement. Such programs should accentuate the need to become involved in an autonomy- and competence-supportive manner, as this study and recent studies (Knollmann and Wild, 2007a,b; Dumont et al., 2014; Gonida and Cortina, 2014; Moroni et al., 2015) have provided evidence of the need to particularly promote the quality rather than quantity of involvement. Hence, teachers should not only learn how to encourage parents to become highly involved; moreover, they should also learn how to assist parents to be more autonomy- and competence-supportive during homework completion. Moreover, parent training programs might help parents to be informed about different parenting styles and their effects on students’ learning and achievement.

### Limitations of the Present Study

First, the generalization of our results is limited due to different attributes of the sample. All analyses were based on parental self-reports. Future studies should assess the study variables by taking other perspectives into account (e.g., school principals, teachers, and students). In these studies, teachers and school principals should be investigated as an additional source of information on EFSC. Their perspectives might differ from parents’ perspectives as teachers and school principals may consider other aspects of EFSC as particularly important than parents. Moreover, in order to improve EFSC in the school, there is a need to identify possible barriers from the school (e.g., teachers’ characteristics) or family (e.g., available time to effectively communicate, etc.) that may undermine teachers’ and parents’ abilities to communicate effectively with each other. Finally, students should rate their well-being in school and at home in future studies. In addition, the generalization of our results is limited due to the high socioeconomic status and the high proportion of mothers in our sample. In our study, the socioeconomic status was not related with parental homework involvement. However, previous studies suggest that high-SES parents tend to be more involved in schooling than other parents. Compared with low-SES parents, their higher education might be associated with feelings of being competent to help leading in higher amounts of involvement (Lee and Bowen, 2006). In the present study, the participants reported on average a comparatively high socioeconomic status. Future studies should take this limitation of the analyzed sample into account and investigate a more representative sample of parents. In future studies, also children with different achievement levels should be considered, as parents of low achieving children or children with special needs might employ other parenting strategies in face of difficulties in school. For these parents and their children, strong FSP might be particularly important. In Germany, cooperation between schools and parents often takes place in the form of short meetings during parent-teacher conferences in school (Sacher, 2008). Commonly, teachers and parents discuss learning problems and children’s grades (Wild and Lorenz, 2010; Yotyodying, 2012). Strong FSP and effective communication might result in a deeper understanding of children’s needs for learning and how parents might support their children’s learning at home. Second, no conclusions on the causality could be drawn due to a cross-sectional research design. Hence, a longitudinal research design should be employed in future studies. Third, the study has exclusively focused on functional ways of parenting (autonomy- and competence-supportive homework involvement), while other parenting styles were not considered here. For instance, according to the SDT perspective on parenting, other forms of parenting such as responsiveness (providing emotional support) and structure (providing clear guidelines and expectations) are related with desired students’ outcomes (for an overview, see Grolnick, 2009) and should thus be analyzed in future studies. Finally, future studies should investigate both qualitative and quantitative ways of parental homework involvement to gain deeper insights into the mechanisms and differences between the two dimensions of involvement.

## Ethics Statement

An ethics approval for this research was not required as per the ethical guidelines of the Faculty of Psychology at FernUniversität in Hagen and regulations of the German Psychological Society due to the noncontroversial nature of the content and the administration of the study. All subjects were parents (adults aged above 21 years). Before their participation, all subjects were informed about the research purposes. Also, they were informed that participation in this research is anonymously and voluntarily. Furthermore, they were informed about the applicable data protection guidelines and the possibility to quit participation whenever they wanted without any disadvantages. Informed consent of the participants was implied through survey completion.

## Author Contributions

SD contributed to the design of the study and the data collection, carried out the analyses and data interpretation, drafted and finalized the manuscript. SY and KJ contributed to the design of the study, parts of the analyses, and data interpretation and provided input for revisions of the manuscript draft.

### Conflict of Interest Statement

The authors declare that the research was conducted in the absence of any commercial or financial relationships that could be construed as a potential conflict of interest.
